# Phospholamban cardiomyopathy: a Canadian perspective on a unique population

**DOI:** 10.1007/s12471-019-1247-0

**Published:** 2019-02-26

**Authors:** C. C. Cheung, J. S. Healey, R. Hamilton, D. Spears, M. H. Gollob, G. Mellor, C. Steinberg, S. Sanatani, Z. W. Laksman, A. D. Krahn

**Affiliations:** 1 Heart Rhythm Services, University of British Columbia, Vancouver, BC Canada; 2 Population Health Research Institute, McMaster University, Hamilton, ON Canada; 3 Division of Cardiology, The Hospital for Sick Children, Toronto, ON Canada; 4 Division of Cardiology, University Health Network, Toronto, ON Canada; 5 Institut Universitaire de Cardiologie et Pneumologie de Québec, Quebec City, QC Canada; 6 Division of Cardiology, BC Children’s Hospital, Vancouver, BC Canada

**Keywords:** Phospholamban, Cardiomyopathy, Sudden cardiac death, Genetics

## Abstract

**Introduction:**

Phospholamban cardiomyopathy is an inherited cardiomyopathy, characterised by a defect in regulation of the sarcoplasmic reticulum Ca^2+^ pump, often presenting with malignant arrhythmias and progressive cardiac dysfunction occurring at a young age.

**Methods:**

Phospholamban R14del mutation carriers and family members were identified from inherited arrhythmia clinics at 13 sites across Canada. Cardiac investigations, including electrocardiograms, Holter monitoring (premature ventricular complexes, PVCs), and imaging results were summarised.

**Results:**

Fifty patients (10 families) were identified (median age 30 years, range 3–71, 46% female). Mutation carriers were more likely to be older, have low-voltage QRS, T‑wave inversion, frequent PVCs, and cardiac dysfunction, compared to unaffected relatives. Increasing age, low-voltage QRS, T‑wave inversion, late potentials, and frequent PVCs were predictors of cardiac dysfunction (*p* < 0.05 for all). Older carriers (age ≥45 years) were more likely to have disease manifestations than were their younger counterparts, with disease onset occurring at an older age in Canadian patients and their Dutch counterparts.

**Discussion:**

Among Canadian patients with phospholamban cardiomyopathy, clinical manifestations resembled those of their Dutch counterparts, with increasing age a major predictor of disease manifestation. Older mutation carriers were more likely to have electrical and structural abnormalities, and may represent variable expressivity, age-dependent penetrance, or genetic heterogeneity among Canadian patients.

**Electronic supplementary material:**

The online version of this article (10.1007/s12471-019-1247-0) contains supplementary material, which is available to authorized users.

## What’s new?

- Among Canadian patients with phospholamban cardiomyopathy, disease manifestations resembled those of their Dutch counterparts, including left ventricular (LV) dysfunction, low-voltage QRS, T‑wave inversion, and frequent premature ventricular complexes.
- Late potentials on signal-averaged ECG were also associated with R14del mutation status and LV dysfunction.
- When stratified by age, older mutation carriers (age ≥45 years old) were more likely to have electrical and structural abnormalities than younger mutation carriers.
- Canadian patients with LV dysfunction were older when compared to the Dutch cohort. These findings may represent variable expressivity, age-dependent penetrance, or genetic heterogeneity in the Canadian population.

## Background

Phospholamban-associated cardiomyopathy is an inherited cardiomyopathy, characterised by a defect in regulation of the sarcoplasmic reticulum Ca^2+^ pump, often presenting with malignant arrhythmias and progressive cardiac dysfunction [1]. Studies have suggested an overlap in clinical features between phospholamban cardiomyopathy, dilated cardiomyopathy, and other rare inherited cardiac conditions, primarily arrhythmogenic right ventricular cardiomyopathy (ARVC) [2].

The phospholamban R14del founder mutation was reported in the Netherlands in a cohort of individuals diagnosed with ARVC or dilated cardiomyopathy (DCM). Individuals expressing the R14del mutation (up to 15% of DCM) were more likely to develop arrhythmic complications and end-stage heart failure than were R14del-negative DCM patients [2]. R14del mutation carriers were also more likely to suffer malignant ventricular arrhythmias at a younger age and die from their disease [3]. Thus, the phospholamban R14del cardiomyopathy portends a much worse prognosis compared to those with ‘typical’ ARVC or DCM, potentially influencing decision-making surrounding advanced heart failure management. In select patients with unexplained familial DCM, genetic testing for phospholamban R14del can be considered in affected individuals and for family screening [4].

Various clinical features are common among R14del mutation carriers on clinical assessment [5]. However, these features often overlap with other cardiomyopathies (e.g. ARVC), and thus, genetic testing is often indicated [6]. All case series to date have reported the Netherlands experience, and we sought to ascertain a Canadian cohort of phospholamban R14del patients. There may be subtle features in standard cardiovascular testing that are unique to Canadian patients. Unique features may allow for earlier identification of disease, prioritise genetic testing, and/or prevention of sudden death in individuals at risk.

## Methods

We performed a retrospective review of Canadian inherited arrhythmia clinics and identified patients and families with the phospholamban R14del mutation. All sites in the Canadian network of inherited arrhythmia clinics, the Hearts in Rhythm Organization (HiRO, www.heartsinrhythm.ca), were contacted to identify eligible patients. All families underwent genetic testing identifying at least one member with the R14del mutation. Data collection included all mutation-positive and mutation-negative surviving family members seen at the clinic. Deceased relatives were not included in this review.

Investigations included baseline 12-lead and high-lead electrocardiogram (ECG), signal-averaged ECG (SAECG), Holter monitor, exercise treadmill testing, echocardiogram, and cardiac magnetic resonance imaging (MRI) results. ECGs were analysed for low-voltage QRS, defined as ≤0.5 mV in all limb and precordial leads. Abnormal T‑wave inversion was defined as inversion ≥0.1 mV below the isoelectric line. Normal parameters for SAECG included filtered QRS duration (<114 ms), low-amplitude signal duration (<38 ms), and root-mean-square terminal 40 ms (>20 uV). Premature ventricular complex (PVC) burden on Holter monitoring was recorded. Echocardiogram and cardiac MRI reports were reviewed for left ventricular (LV) dysfunction. The study was approved by the local institutional research ethics boards at each contributing centre.

### Statistical analysis

Statistical analysis focused on surviving patients where electrocardiographic and imaging data were available. The population was divided into R14del mutation carriers and unaffected relatives, and mutation carriers were subdivided based on median age: <45 years and ≥45 years. Fisher’s exact test was used to compare electrocardiographic (ECG, SAECG, Holter) and structural abnormalities between groups. Mann-Whitney U test was used to compare the age and overall burden of PVCs between groups. Univariate logistic regression analysis was performed to identify various associations with LV dysfunction among mutation carriers.

## Results

Thirteen sites across Canada were contacted, and cases were identified at 5 centres. Fifty individuals from 10 families were screened, including 34 mutation carriers, 14 non-carrier relatives, and 2 patients with genetic testing in progress. One individual passed away during her fifth decade of life after cardiac transplantation. Among surviving patients, the median age was 30 years (range 3–71 years), with 46% female (23 out of 50). Families were enrolled in British Columbia (19 out of 50) or Ontario (31 out of 50).

### R14del-positive patients

Genetic testing identified 34 individuals (68%, out of 50 surviving individuals) with the phospholamban R14del mutation. A detailed report of disease manifestations among surviving mutation carriers is provided in Supplementary Tab. 1. Mutation carriers were more likely to be older, have ECG abnormalities including low-voltage QRS and T‑wave inversion, and frequent PVCs, compared to unaffected relatives. Structural abnormalities were also more common among mutation carriers than in unaffected relatives (Tab. 1). Exercise testing was performed infrequently in the cohort (*n* = 11; Tab. 1).

**Table 1:** Patient characteristics by R14del mutation status

Characteristic	R14del-positive (*n* = 34)	R14del-negative (*n* = 14)	*p*-value
Age, median (IQR)	32.5 (29, 56)	25 (17, 42)	** 0.011**
Sex, female (%)	16 (47)	7 (50)	0.85
Low-voltage QRS (%)	13 (59)	0 (0)	**<0.001**
T-wave inversion (%)	10 (53)	0 (0)	** 0.002**
*Signal-averaged ECG*			
– Filtered QRS duration, ms (IQR)	108 (103, 113)	110 (104.5, 113.5)	0.57
– Low-amplitude signal, ms (IQR)	36 (31, 48)	31.5 (20.5, 36.5)	** 0.019**
– Root-mean-square, µV (IQR)	26 (15, 35)	38.5 (26.5, 44)	0.051
– Late potentials, ≥1 (%)	11 (48)	4 (33)	0.41
*Holter monitoring*			
– PVCs, median (IQR)	420 (2, 963.5)	0.5 (0, 1)	** 0.004**
– > 500 PVCs (%)	12 (43)	0 (0)	** 0.046**
*Exercise treadmill testing*			
– PVCs during exercise (%)	2 (33)	1 (33)	1.00
– Ventricular tachycardia (%)	2 (29)	0 (0)	0.30
LV dysfunction (%)	10 (33)	0 (0)	** 0.017**

*IQR* interquartile range, *PVC* premature ventricular complex, *LV* left ventricular

**Fig. 1:**
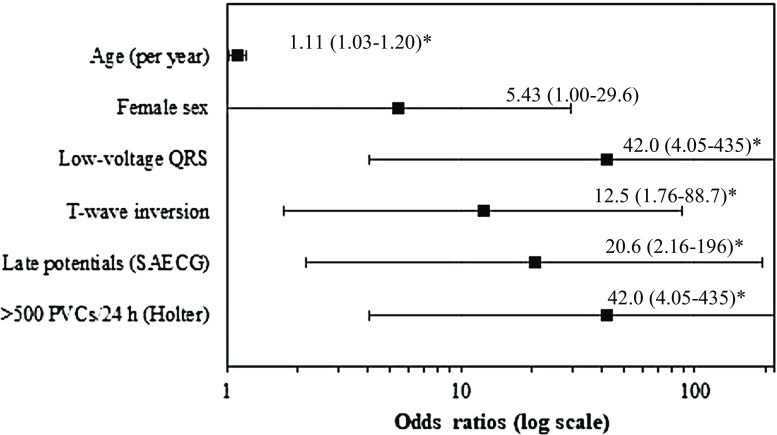
Forest plot of predictors of left ventricular dysfunction in R14del-positive patients. (^*^Statistical significance including age (*p* = 0.004), low-voltage QRS (*p* = 0.002), T‑wave inversion (*p* = 0.012), late potentials (*p* = 0.009), and frequent premature ventricular complexes (*PVCs*) (*p* = 0.002). *SAECG* signal-averaged ECG)

Among mutation carriers, LV dysfunction was associated with increasing age, low-voltage QRS, T‑wave inversion, SAECG late potentials, and frequent PVCs. These findings are illustrated in Fig. 1 and Tab. 2.

**Table 2:** Various associations with left ventricular dysfunction in R14del-positive patients

Characteristic	Odds ratio (95% CI)	*p*-value
Age (per year)	** 1.11 (1.03–1.20)**	**0.004**
Female sex	5.43 (1.00–29.6)	0.051
Low-voltage QRS	**42.0 (4.05–435)**	**0.002**
T-wave inversion	**12.5 (1.76–88.7)**	**0.012**
*Signal-averaged ECG*		
– Filtered QRS duration (per ms)	1.02 (0.97–1.08)	0.426
– Low-amplitude signal (per ms)	** 1.21 (1.04–1.41)**	**0.015**
– Root-mean square (per µV)	** 0.88 (0.80–0.97)**	**0.012**
– Late potentials, ≥1 (%)	**20.6 (2.16–196)**	**0.009**
*Holter monitoring*		
– Per PVC	** 1.00 (1.00–1.00)**	**0.018**
– > 500 PVCs over 24 h	**42.0 (4.05–435)**	**0.002**

*CI* confidence interval, *PVC* premature ventricular complex

**Bold** indicates *p* < 0.05

### Older patients and outcomes

When stratified by age, older mutation carriers (age ≥45 years old) were more likely to have electrical and structural abnormalities than younger mutation carriers (Tab. 3). This included ECG abnormalities (low-voltage QRS), SAECG late potentials, frequent PVCs, and LV dysfunction. The median age of patients with LV dysfunction was 56 years (range 45–61).

**Table 3:** Comparison of R14del-positive patients by age

Characteristic	Age >45 years (*n* = 16)	Age <45 years (*n* = 18)	*p*-value
Age, median (IQR)	56.5 (54, 59.5)	29 (24, 30)	**<0.001**
Sex, female (%)	9 (56)	7 (39)	0.31
Low-voltage QRS (%)	11 (92)	2 (20)	**<0.001**
T‑wave inversion (%)	7 (70)	3 (33)	0.11
*Signal-averaged ECG*			
– Filtered QRS duration, ms (IQR)	108 (104, 113)	108 (100, 112)	0.58
– Low-amplitude signal, ms (IQR)	46 (42, 53)	34 (28.5, 35.5)	**0.007**
– Root-mean-square, µV (IQR)	15 (11–23)	33 (26.5, 45)	**0.005**
– Late potentials, ≥1 (%)	9 (82)	2 (17)	**0.002**
*Holter monitoring*			
– PVCs, median (IQR)	882.5 (470, 2653)	2 (1, 370)	**<0.001**
– > 500 PVCs (%)	9 (64)	3 (21)	**0.022**
LV dysfunction (%)	10 (67)	0 (0)	**<0.001**

*IQR* interquartile range, *PVC* premature ventricular complex,* LV* left ventricular

### Follow-up events and Dutch comparison

Two patients underwent cardiac transplantation, including one patient who suffered a cardiac arrest with persistent severe LV dysfunction, and one patient who is now deceased after cardiac transplantation (data not available). When compared to the original Dutch cohort (mean age of presentation 44.3 ± 12.6 years) [2], Canadian patients with LV dysfunction were older (*p* = 0.015). Five patients (all mutation carriers) received implantable cardioverter defibrillators (ICDs), including one patient with recurrent polymorphic ventricular tachycardia. One patient suffered syncope during physical activity, and subsequently passed away due to ventricular tachycardia storm.

## Discussion

We reported the findings in a Canadian population of phospholamban R14del mutation carriers and unaffected family members. This is the first report of descendants from the original Dutch founder population of patients with phospholamban R14del cardiomyopathy [2]. In this study, mutation carriers were more likely to be older and have ECG changes (low-voltage QRS, T‑wave inversion), frequent PVCs, and LV dysfunction compared to their unaffected relatives. Increasing age, low-voltage QRS, T‑wave inversion, late potentials, and frequent PVCs were predictors of LV dysfunction among mutation carriers, with disease onset occurring at an older age compared to their Dutch counterparts.

The cellular basis for phosholamban cardiomyopathy has been well described [7–10]. Clinical reports were first published in large cohorts in the Netherlands, with the phospholamban R14del mutation identified in up to 15% of patients diagnosed with ARVC or DCM [2]. R14del mutation carriers had a low-voltage ECG (46%), and a malignant phenotype, including appropriate ICD discharges (47% vs 10%), family history of SCD (36% vs 16%), and end-stage heart failure requiring transplantation (36% vs 16%) [2]. In a subsequent evaluation of outcomes, van Rijsingen et al. followed 403 phospholamban R14del mutation carriers for a median period of 42 months. The authors identified a significant excess in mortality starting from 25 years old, and a standardised mortality ratio of 1.7 (95% confidence interval 1.4–2.0) [3]. R14del mutation carriers frequently had malignant ventricular arrhythmias (19%) and end-stage heart failure (11%), occurring as early as 20 years and 31 years, respectively [3]. Malignant arrhythmias were more common in patients with LV dysfunction and/or a history of sustained or non-sustained arrhythmias.

Our Canadian cohort of patients with phospholamban cardiomyopathy was similar to the original Dutch founder population, with some important differences. Findings of low-voltage QRS and LV dysfunction were seen across both populations and represent the common manifestations of phospholamban cardiomyopathy. Specifically, a low-voltage QRS may serve as an important discriminator when evaluating the baseline ECG [5]. Previous studies have demonstrated that phospholamban mutation carriers with T‑wave inversion were more likely to have left ventricular late gadolinium enhancement on cardiac MRI, compared to those without T‑wave inversion. In our cohort, we demonstrated a strong association between T‑wave inversion and LV dysfunction among mutation carriers [11]. Furthermore, fibrosis and fatty changes among phospholamban carriers may also occur in a distinct pattern compared to other forms of hereditary cardiomyopathies [12]. In our cohort, we identified a trend towards late potentials on SAECG, which may represent a novel finding in phospholamban cardiomyopathy patients and requires validation in other cohorts. Late potentials likely reflect the underlying disease process in phospholamban cardiomyopathy that leads to progressive LV dysfunction (i.e. progressive fibrosis leading to depolarisation dispersion, manifested as late potentials) [13, 14].

When we stratified our analysis by age, older mutation carriers were more likely to have disease manifestations, including low-voltage QRS, late potentials, and frequent PVCs, with younger patients having few or no disease manifestations. Furthermore, a significantly greater proportion of older patients had LV dysfunction compared to their younger counterparts, with LV dysfunction occurring at a median age of 56 years (range 45–61 years). Two patients underwent cardiac transplantation. End-stage heart failure occurred as early as 31 years in the Dutch cohort, with up to 11% of patients suffering from end-stage heart failure [3]. Canadian R14del mutation carriers may potentially present later in life than their Dutch counterparts. Further studies would be required to understand whether the delayed manifestation of the phospholamban phenotype may be the result of genetic heterogeneity, when most studies suggest a Mendelian inheritance of the phospholamban cardiomyopathy phenotype [5].

Limitations of our study included our small sample size relative to the Dutch cohort. We had only a small number of cardiovascular outcomes, including end-stage heart failure and arrhythmic events. This limited the extent of our statistical analyses, and only univariate analyses were performed. Most of the risk markers identified have been previously reported, although late potentials are a novel finding. We presented the first study of descendants of the Dutch phospholamban R14del founder population, and reported both consistent and novel findings among Canadian patients.

## Conclusions

A Canadian cohort of patients with the phospholamban R14del mutation had a malignant course with frequent biventricular cardiomyopathy and ventricular arrhythmias. Canadian patients had similar expression of disease to the original Dutch cohort, with older mutation carriers more likely to have electrical disease and structural abnormalities compared to younger mutation carriers. These findings may represent variable expressivity, age-dependent penetrance of the R14del mutation, or genetic heterogeneity of the Canadian population.

## Caption Electronic Supplementary Material

Supplementary Table 1 Disease manifestations among R14del-positive patients
