# Supplementary material for: Phospholamban cardiomyopathy: a Canadian perspective on a unique population
Source: Neth Heart J. 2019 Feb 26;27(4):208–13. doi: 10.1007/s12471-019-1247-0 (PMC6439019; doi:10.1007/s12471-019-1247-0)
Supplement: Supplementary file 1 — Supplementary Table 1 Disease manifestations among R14del-positive patients [file 12471_2019_1247_MOESM1_ESM.docx]

**SUPPLEMENTAL TABLES**

**Supplemental Table 1. Disease Manifestations among R14Del-positive Patients**

| **Patient #** | **Age (in years)** | **Sex** | **Low-Voltage QRS** | **T-wave Inversion** | **SAECG (Late Potentials)** | **Holter (PVCs/24 hours)** | **LV Dysfunction (on Echo)** |
| --- | --- | --- | --- | --- | --- | --- | --- |
| 1 | 11 | Male | No | No | No | 0 | No |
| 2 | 12 | Female | No | No | No | 1 | No |
| 3 | 12 | Male | No | No | No | N/A | No |
| 4 | 16 | Female | No | Yes | No | 3 | No |
| 5 | 24 | Female | N/A | N/A | No | 1 | No |
| 6 | 26 | Female | No | Yes | No | 48 | No |
| 7 | 28 | Male | N/A | N/A | N/A | N/A | N/A |
| 8 | 28 | Male | N/A | N/A | No | 986 | N/A |
| 9 | 29 | Female | No | No | No | 2 | No |
| 10 | 29 | Male | No | No | N/A | 763 | No |
| 11 | 29 | Male | Yes | Yes | Yes | 3 | No |
| 12 | 29 | Female | N/A | N/A | No | 0 | No |
| 13 | 30 | Male | N/A | N/A | N/A | N/A | N/A |
| 14 | 30 | Male | N/A | N/A | N/A | N/A | N/A |
| 15 | 31 | Male | Yes | N/A | Yes | 370 | No |
| 16 | 31 | Male | N/A | N/A | N/A | 2 | No |
| 17 | 32 | Male | No | No | N/A | 763 | No |
| 18 | 33 | Female | N/A | N/A | No | 1 | No |
| 19 | 45 | Female | Yes | Yes | Yes | 824 | Yes |
| 20 | 47 | Male | Yes | Yes | No | N/A | N/A |
| 21 | 48 | Female | Yes | Yes | Yes | 243 | Yes |
| 22 | 54 | Male | No | No | Yes | 2816 | Yes |
| 23 | 54 | Female | Yes | Yes | Yes | 1878 | Yes |
| 24 | 56 | Female | Yes | No | N/A | N/A | Yes |
| 25 | 56 | Female | N/A | N/A | Yes | 941 | Yes |
| 26 | 56 | Female | Yes | N/A | Yes | 760 | Yes |
| 27 | 57 | Female | Yes | Yes | No | 3675 | Yes |
| 28 | 58 | Male | Yes | Yes | Yes | 5028 | Yes |
| 29 | 58 | Male | N/A | N/A | N/A | 470 | No |
| 30 | 59 | Male | Yes | Yes | N/A | 2653 | No |
| 31 | 60 | Male | N/A | N/A | N/A | 53 | No |
| 32 | 61 | Female | Yes | N/A | Yes | 1042 | Yes |
| 33 | 62 | Male | N/A | N/A | Yes | 223 | No |
| 34 | 71 | Female | Yes | No | N/A | 488 | No |
